# Ruptured isolated spinal artery aneurysms: a rare manifestation of an arterial dissecting disease

**DOI:** 10.3389/fneur.2025.1567536

**Published:** 2025-05-29

**Authors:** T. P. Kee, T. Krings, J. Pace, S. K. Swaminathan, E. Orru'

**Affiliations:** ^1^Department of Neuroradiology, National Neuroscience Institute, Singapore, Singapore; ^2^Division of Neurointerventional Radiology, Lahey Hospital & Medical Center - Beth Israel Lahey Health, UMass Chan Medical School, Boston, MA, United States; ^3^Department of Medical Imaging, University of Toronto, Toronto, ON, Canada; ^4^Edward Singleton Department of Radiology, Texas Children's Hospital, Baylor College of Medicine, Houston, TX, United States; ^5^Division of Neurosurgery, Lahey Hospital & Medical Center - Beth Israel Lahey Health, UMass Chan Medical School, Boston, MA, United States

**Keywords:** spinal aneurysm, spinal dissection, arterial dissection, spinal hemorrhage, spinal vascular malformation

## Abstract

Isolated spinal artery aneurysms (ISAAs) are a rare cause of intracranial and spinal hemorrhages with unclear pathophysiology and natural history and non-standardized management strategies. We hereby present two cases of ruptured ISAAs of posterior spinal arteries treated with open surgery and embolization, respectively. Case presentations are followed by a comprehensive literature review on ISAA pathophysiology, natural history, and management strategies.

## Introduction

Subarachnoid hemorrhage (SAH) from a ruptured isolated spinal artery aneurysm (ISAA) is a very rare entity (1). While ISAAs are believed to be dissecting in nature (1, 2), their exact pathophysiology and natural history have yet to be clarified, as the condition has mostly been described in case reports or small series. High heterogeneity in clinical presentation and management strategies, as well as scarcity of pertinent data from the existing literature results in the absence of standardized diagnostic and treatment guidelines. We report two cases of ruptured ISAAs of posterior spinal arteries (PSAs) and present a literature review with particular attention to imaging features and treatment options for this rare condition.

## Methods

We included two cases of ruptured ISAAs from our institutions and performed a literature review on the topic. A PubMed literature search was performed using the terms “spinal”, “artery”, “aneurysm”, “subarachnoid”, “h(a)emorrhage”, and/or combinations thereof. Titles and abstracts were screened for relevance by the first author. Case reports, case series, and review articles that specifically discussed cases of ruptured ISAAs were included. To ensure that reports were not overlooked, we reviewed the references of each relevant article to identify secondary sources for inclusion. Cases of intra- or perinidal spinal arterial aneurysms associated with vascular malformations were excluded. For reports published in 2010 and earlier, information was extracted from the literature review by Kim HJ and Choi IS in 2011, which summarized 43 cases from 38 reports. Two additional case reports published before 2010 found in our literature search were also added to the case analysis, summarized in Table 1 and referenced accordingly. For reports published after 2010, all cases are summarized in Table 1, and individual cases are summarized in Table 2.

**Table 1:** Summary of case studies and case series from 1930 to 2010 (40 articles) and 2011 to 2024 (45 articles).

**Year of publications/no. of studies and cases**	**Age/sex**	**Artery involved**	**Spinal level**	**Treatment**	**Outcome**
2010 and earlier (40 studies, 45 cases) (2, 3, 15, 21, 23–25, 27, 28, 33, 35–62)	Mean age 55 29 F 16 M	26 ASA 9 PSA 4 LSA 6 unknown	24 C 17 T 4 TL	26 Surgery 2 Embolization 14 Conservative 3 Unknown	33 clinical improvement 9 deaths (5 conservative, 1 surgery, 1 embolization, 2 unknown) 3 unknown
2011-2024 (45 studies, 70 cases)	Mean age 56 40F 30M	38 ASA 25 PSA 4 LSA 3 unspecified	22 C 32 Th 5 TL 11 Unknown levels	24 Surgery 13 Embolization 26 Conservative 7 Unknown Tx	60 clinical improvement 2 deaths (both conservatives) 8 unknown

anterior spinal artery (ASA), posterior spinal artery (PSA), lateral spinal artery (LSA), cervical (C), thoracic (T), lumbar (L), male (M), and female (F).

**Table 2:** Summary of all published reports on isolated spinal artery aneurysms from 2011 to 2024.

**Author, year**	**Age, y/sex**	**Clinical presentation**	**Imaging findings**	**Treatment**	**Outcome**
Iihoshi et al. 2011 (63)	60/F	Headache and back pain	Posterior fossa and spinal SAH T12 radiculomedullary artery aneurysm	Conservative—Spontaneous occlusion with preserved ASA	Clinical improvement
Kim and Choi 2012 (1)	52/M	Abdominal and back pain	Spinal SAH T8 PSA aneurysm	Embolization (coils and NBCA)	Cure Clinical improvement
Sato et al. 2012 (34)	67/F	Back pain, paresthesia, sphincter disturbance	Spinal SAH and cord infarction T11 radiculomedullary and T8 radiculopial aneurysms	Conservative—Spontaneous occlusion	Clinical improvement
Morigaki et al. 2012 (12)	78/M	Tetraparesis and LOC	SAH LSA aneurysm at the C2 level	Embolization (coils)	Partial PICA infarct Clinical improvement
Van Es et al. 2013 (58) (Case 1)	62/F	Headache Back pain	Spinal SAH Left L1 radiculopial aneurysm	Surgery (aneurysm resection)	NA
Van Es et al. 2013 (58) (Case 2)	68/M	Headache and back pain	Spinal SAH T4 radiculopial aneurysm	NA	NA
Son et al. 2013 (64)	45/F	Headache and back pain	Intracranial and spinal SAH Art. of AdamKwz ASA T12 aneurysm	Conservative—Spontaneous occlusion	Clinical improvement
Yang 2013 (26)	47/M	LOC	SAH ASA (from Rt Vert) dissecting aneurysm	Conservative—no change to aneurysm, no rebleed	Died @103 days post-SAH (cancer-related)
Marovic et al. 2013 (65)	58/M	Back pain	Spinal SAH T3 radicular spinal artery aneurysm	Surgery (aneurysm resection)	Cure Unknown clinical outcome
Romero et al. 2014 (11)	37/F	Chest pain, back pain, headache	Post fossa SAH T3 radiculomedullary aneurysm	Conservative—Spontaneous occlusion	Clinical improvement
Romero et al. 2014 (11)	72/F	Neck and back pain	Intracranial and spinal SAH T11 radiculopial aneurysm	Conservative—nil imaging f/up	Clinical improvement
Pahl et al. 2014 (66)	43/F	Headache, vomiting, LOC	SAH and IVH ASA (from Lt Vert) aneurysm	Conservative—Spontaneous occlusion	Clinical improvement
Horio et al. 2015 (4)	84/M	Altered mental status	SAH T12 Radiculopial artery aneurysm	Surgery (aneurysm resection)	Cure Clinical improvement
Ronchetti et al. 2015 (32) (Case 1)	51/F	Headache, neck pain, lower limb numbness	Posterior fossa SAH, spinal SAH and SDH 2 thoracic PSA aneurysms in one patient	Surgical resection (aneurysm resection cx wound infection)	Cure Clinical improvement
Ronchetti et al. 2015 (32) (Case 2)	68/M	Headache, back pain	Posterior fossa and spinal SAH Thoracic PSA aneurysm	Embolization (with particles)	Cure Clinical improvement
Ashour et al. 2015 (18)	72/M	Headache	Cranial SAH C2 ASA aneurysm (bilateral VA occlusions)	Surgery (aneurysm clipping and wrapping)	Cure Clinical improvement
Nakhla et al. 2016 (8)	88/F	Headache and neck pain	Cranial and upper spinal SAH Cervical ASA aneurysm (related to a herniated disk)	Conservative	Clinical improvement
Ikeda et al. 2016 (29)	54/M	Back pain and vomiting	Spinal SAH and SDH T10 radiculopial artery aneurysm	Surgery (aneurysm resection)	Cure Clinical improvement
Doberstein et al. 2016 (67)	59/M	Back pain and lower limb weakness	SAH T11 Art. Of AdamKwz (ASA) aneurysm	Conservative	Clinical improvement
Takata et al. 2016 (68)	72/M	Back pain	Spinal SAH T9 radiculopial artery	Surgery (aneurysm resection)	Cure Clinical improvement
Agarwal et al. 2016 (69)	47/F	Back pain	Rt T10 intercostal artery aneurysm, close to the origin of Art. Of AdamKwz	Conservative—Spontaneous occlusion	Clinical improvement
Aguilar-Salinas et al. 2017 (70)	54/F	Backpain and headache	Spinal SAH Left T10 Art. Of AdamKwz (ASA) aneurysm	Conservative—Spontaneous occlusion	Clinical improvement
Ren et al. 2017) (71) (Case 1)	57/F	Headache	SAH C1 ASA aneurysm	Surgery (aneurysm resection)	Cure Clinical improvement
Ren et al. 2017 (71) (Case 2)	27/F	Lower limb pain and numbness	L2 RMA aneurysm	Surgery (aneurysm resection)	Cure Clinical improvement
Singh et al. 2017 (13) (Case 1)	18/M	Severe neck pain and UL weakness	SAH C7 ASA aneurysm (coarctation of the aorta)	Conservative	Unknown outcome
Singh et al. 2017 (13) (Case 2)	25/F	Severe back pain with paraplegia	SAH T5 ASA aneurysm (Takayasu arteritis)	Conservative	Unknown outcome
Morozumi et al. 2017 (14)	9/M	Back pain and gain disturbance	SAH C7-T1 intramedullary aneurysm artery (histology: few inflammatory cells)	Surgery (aneurysm resection)	Cure Clinical improvement
Dabus et al. 2018 (72) (4 pts)	Mean age 63/2F, 2M	2 Back pain and sensory deficits 1 Back pain only 1 Head and neck pain	Spinal hemorrhage (2 SAHs, 1 small intramedullary haematoma, 1 SDH) 2 ASA (AMA) aneurysms 2 PSA aneurysms 2 cervical, 2 thoracic	Conservative—Spontaneous occlusion	Clinical improvement
Renieri et al. 2018 (10) (Case series of 11 patients)	Mean age 60 7F, 4M	9 Back pain 7 LL weakness	Mostly SAH, 2 with SDH 3 ASA aneurysms (radiculomedullary) 8 PSA aneurysms (radiculopial) (level not specified)	5 Embolization (2 with particles, 3 with coiling) 2 Surgery (1 surgical trapping, 1 aneurysm resection) 4 Conservative	Mostly occluded aneurysms with clinical improvement 3 had no f/up
Simon-Gabriel et al. 2018 (17)	65/M	Drowsy with neck stiffness	SAH Proximal ASA aneurysm arising from VA	Embolization (Flow-diverter)	Regressed aneurysm with clinical improvement
Aljuboori et al. 2018 (73)	78/M	Back pain and lower limb weakness	T9 Artery of Adamkiewicz aneurysm	Surgery (aneurysm clipping)	Cure Clinical improvement
Roka 2019 (74)	30/F	Headache and vomiting	Cervical ASA aneurysm	Conservative	Clinical improvement
Priola et al. 2019 (75)	54/F	Upper back pain	Spinal SDH and SAH T3 radiculomedullary artery aneurysm	Surgery (aneurysm resection)	Cure Clinical improvement
Yokosuka et al. 2019 (76)	79/F	Headache and vomiting	SAH T10 radicular artery aneurysm	Surgery (aneurysm resection)	Cure Clinical improvement
Hanakita et al. 2019 (77)	77/NA	N/A	Posterior fossa SAH ASA originating from the right VA	Surgery (extirpation of aneurysm with bipolar coagulation)	Cure Clinical resolution
Nguyen et al. 2020 (78)	45/M	Abdominal pain, Severe headache, seizure, paraparesis	Spinal SAH, SDH and cord compression T9 radiculomedullary artery	Surgery (aneurysm resection)	Cure Stable neurology
Cobb et al. 2020 (9)	36/F	Acute low back pain and paraplegia	T3-L5 SDH and spinal cord infarct of the lower TL cord T11 ASA (radiculomedullary) aneurysm	Embolization (Onyx)	Cure Stable neurology
Takebayashi et al. 2020 (79)	67/F	Back pain	Intracranial SAH T10 radiculopial	Surgery (aneurysm resection)	Cure Clinical improvement
Abdalkader et al. 2021 (22)	2M (50s, 70s) 2F (40s, 60s)	3 headaches 1 confusion	2 ASA 2 radiculomedullary 3 Cervical 1 Thoracic	1 Surgery (clipping) 3 Conservative	3 improved 1 (conservative) died of vasospasm
Limaye et al. 2021 (80)	43/F	Low back pain and paraesthesia	Spinal SAH and SDH T12 (supplied by L2) ASA aneurysm	Conservative—Spontaneous occlusion	Clinical improvement
Crobeddu et al. 2021 (81)	62/M	Acute pain in the crural -thigh region	Spinal SAH L1 radiculopial aneurysm	Not found on surgery; repeat DSA-occluded	Clinical improvement
Bergeron 2021 (7) (4 isolated cases)	Mean age 52/F	Headache and back pain	Intracranial and spinal SAH 1 radiculomedullary 3 radiculopial All 4 thoracic	1 Surgery (aneurysm resection) 3 Conservative	Cure Clinical improvement
Shima et al. 2021 (19)	77/F	Headache and drowsiness	Cranial SAH C4 ASA aneurysm (bilateral VA occlusions)	Endovascular (aneurysm coiling and VA stenting)	Cure Clinical improvement
Gomez et al. 2023 (82)	Adult/F	Low back pain	Spinal epidural hematoma and SAH (L1-L4 levels) L2 radiculomedullary	Conservative—Spontaneous occlusion	Clinical improvement
Liu et al. 2023 (83)	64/M	Headache and vomiting	Post fossa and upper cervical SAH C4 radiculomedullary artery aneurysm	Conservative—Spontaneous occlusion	Clinical resolution
Jeon et al. 2024 (31)	51/F	Severe headache, neck pain	Posterior fossa SAH LSA aneurysm from PICA	Embolization	Cure Clinical improvement
Ha et al. 2024 (30)	52/M	Acute lower back pain and bil. Leg pain	T12-L1 radiculomedullary (ventral lateral aspect of spine)	Surgery (resection of thrombosed aneurysm)	Cure Clinical improvement
Papadimitriou et al. 2024 (84)	74/F	Headache	Post fossa SAH LSA aneurysm	Surgery (aneurysm resection)	Cure Clinical improvement
Song et al. 2024 (85) (3 patients)	Mean age 64/2M, 1F	Headache	Post fossa SAH LSA aneurysms	2 Surgery (aneurysm clipping) 1 lost to f/up	Cure Clinical improvement
Zhao and Yu 2024 (16)	62/M	Headache	Post fossa SAH Aneurysm along collateral circulation from chronically occluded distal V4 VA supplying ASA	Embolization (flow-diverter)	Occluded flow-diverter with partial PICA infarcts regressed aneurysm

Subarachnoid hemorrhage (SAH), subdural hemorrhage (SDH), loss of consciousness (LOC), anterior spinal artery (ASA), posterior spinal artery (PSA), lateral spinal artery (LSA) male (M), female (F), posterior inferior cerebellar artery (PICA), and vertebral artery (VA).

## Case reports

### Case 1

A 60-year-old woman with a history of hypertension, dyslipidemia, and diabetes was admitted for workup of acute-onset chest pain after an episode of abdominal pain with vomiting and diarrhea. The next day, she developed acute lower limb weakness and numbness, along with urinary retention. On examination, she was alert, with a Glasgow Coma Scale score of 15. Power of bilateral upper extremities was 5/5 at the C5 level, 3/5 at C6–C7 levels, and 1/5 at the C8-T1 levels, with flaccid paresis (power 0/5) of bilateral lower extremities. She had absent sensation at the sensory level of T2 and below, along with saddle anesthesia.

**Figure 1:**
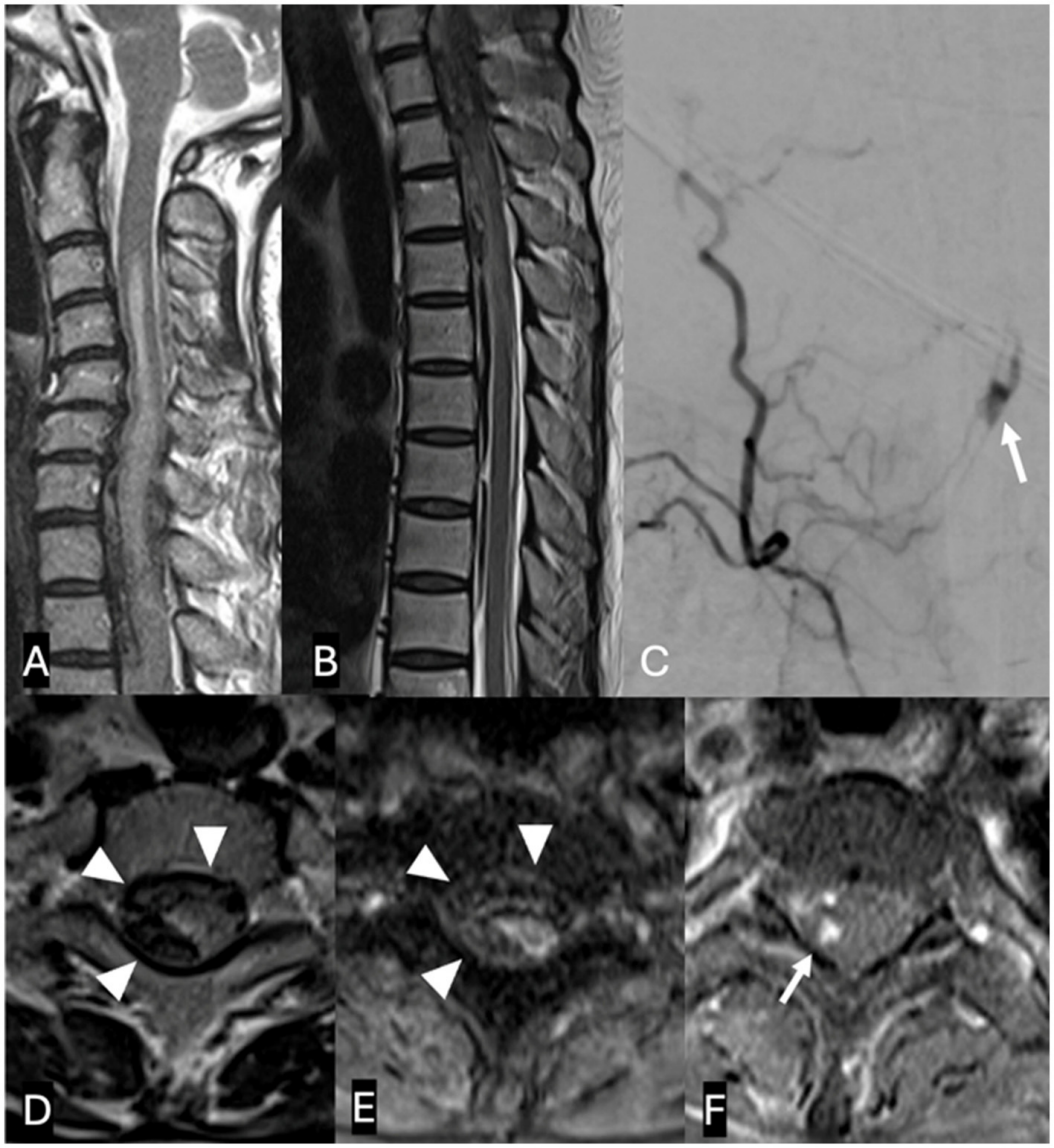
A 60-year-old woman presented with acute back pain and flaccid quadriparesis. A, B: Sagittal T2-weighted MRI images of the spine [**(A)** cervical spine and **(B)** thoracic spine] show a subdural hematoma extending along the lower cervical and upper thoracic spine, and spinal subarachnoid hemorrhage extending down to the level of T9. Note associated cord edema. **(D, E)** Axial T2-weighted **(D)** and gradient echo **(E)** images demonstrate the SDH centered predominantly along the right side of the spinal canal (arrowheads), displacing the cord to the left. **(F)** Postcontrast T1-weighted image shows focal nodular enhancement within the subdural hematoma (**arrow**), corresponding to a fusiform “tent-like” dissecting aneurysm arising from the right T2 radiculopial artery on spinal angiogram (**C**, **arrow**). This was surgically resected and characterized by histology as an organizing hematoma.

Magnetic resonance imaging (MRI) of the spine (Figure 1) demonstrated a subdural hematoma with spinal cord edema along the lower cervical and upper thoracic spine, with SAH tracking down to T9 level. On postcontrast T1-weighted sequences, there was focal nodular enhancement within the hematoma at the level of T2. The patient underwent a spinal angiogram, which demonstrated a fusiform dilatation of the right PSAs originating at the T2 level consistent with an ISAA.

Given significant cord compression, the patient underwent a C7-T2 laminectomy with evacuation of the spinal hematoma; upon surgical exploration, there was no evidence of a spinal abscess. At the time of decompression, PSAs originating from the right T2 intersegmental artery was clipped, and the aneurysm was excised. Pathology demonstrated a disrupted vessel wall with surrounding organized hematoma in keeping with a dissecting aneurysm of the PSAs. No evidence of neutrophilic infiltration on pathology. The cause was thought to be a traumatic dissection, potentially resulting from significant abdominal hypertension from vomiting and diarrhea.

Over the following week, the patient developed septic shock requiring resuscitation and intubation. Abdominal imaging showed evidence of acute descending colitis, pyelonephritis, and a renal abscess. Blood cultures were positive for *Bacteroides uniformis*, likely from the gastrointestinal tract, and *Morganella morganii*. After successful treatment, the patient was discharged to rehab, where she recovered almost complete upper limb but remained paraplegic, with neurogenic bladder and bowel. Follow-up imaging of the spine was not performed.

### Case 2

A 66-year-old woman with a history of mechanical aortic valve replacement and atrial fibrillation on warfarin, hypertension, and dyslipidemia, presented with acute left lower extremity weakness. A few days prior to admission, she had started noticing back pain radiating to the left lower extremity after picking up a crate at work. Over the following days, the pain gradually worsened, with an acute exacerbation the night prior to admission, which was followed by unsteadiness and significant left lower extremity weakness. On examination, the patient had significant weakness in left hip flexion and numbness in the left lower extremity.

**Figure 2:**
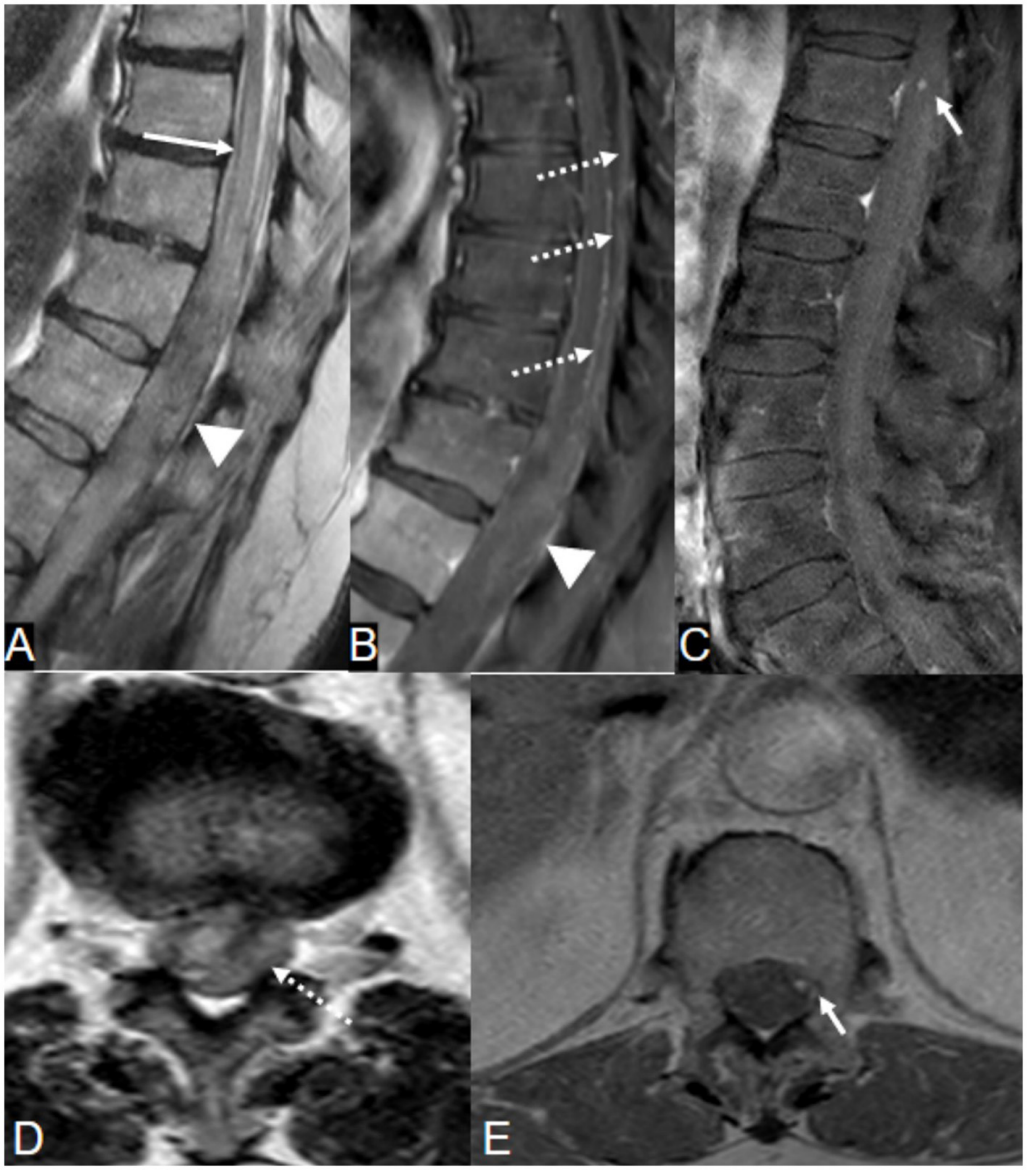
A 66-year-old woman on warfarin presented with acute severe low back pain. **(A, B, D)** T2-weighted MRI images demonstrate conus edema (long arrow) with spinal subdural hematoma, predominantly on the left, displacing the conus to the right (arrowheads). Note the lateral compression of the spinal cord and cerebrospinal fluid by the hematoma (long arrowhead). **(C, E)** Contrast-enhanced MRI shows focal nodular enhancement at the left peripheral aspect of the cord at the T12 level (short arrow), confirmed later on angiogram as a ruptured left T12 posterior spinal artery dissecting aneurysm (Figure 3A).

**Figure 3:**
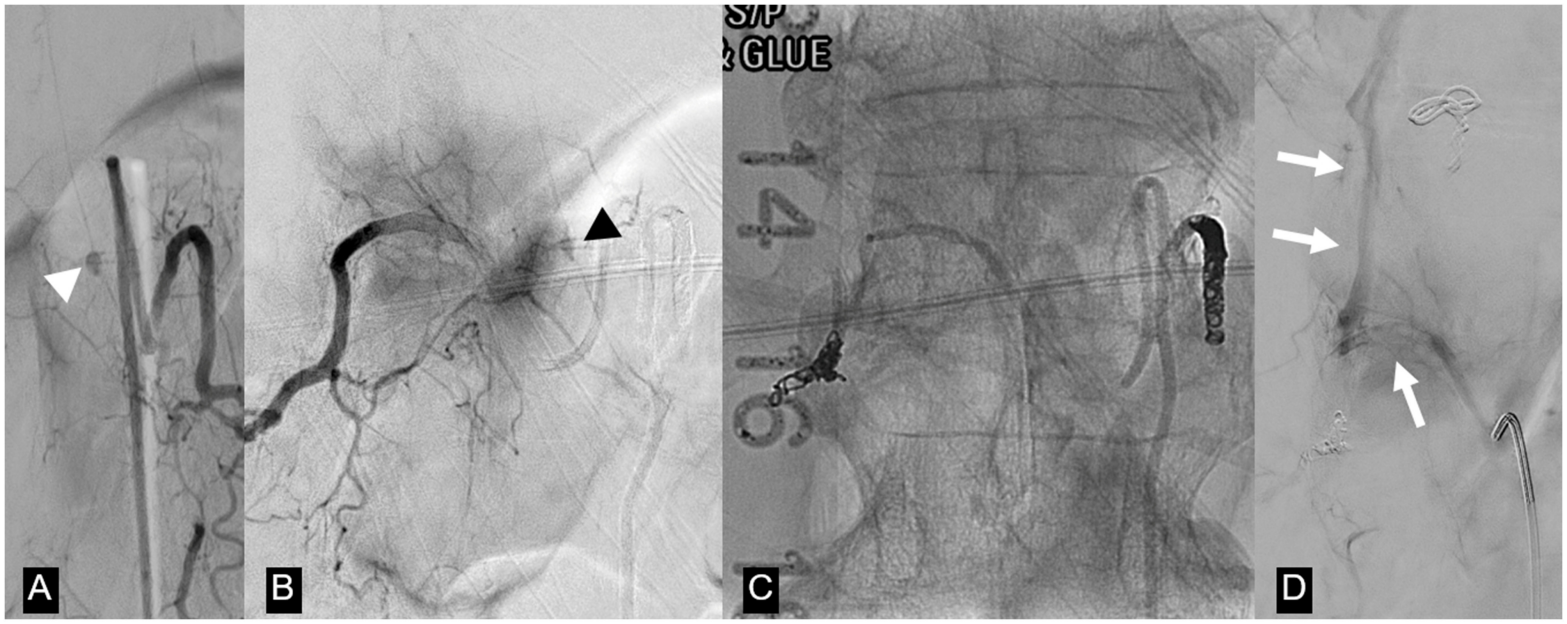
Same patient as in Figure 2. **(A)** Left T12 intersegmental artery (ISA) angiogram demonstrates an oval-shaped dissecting pseudoaneurysm arising from a posterior spinal artery (white arrowhead). **(B)** Right T12 ISA angiogram postcoil embolization of the left T12 ISA shows retrograde filling of the pseudoaneurysm through retrocorporeal collaterals (black arrowhead). **(C)** Unsubtracted image of the T12 vertebral body demonstrates coil and n-butyl cyanoacrylate occlusion of bilateral T12 ISAs. **(D)** Repeat T12 ISA angiogram obtained after the deterioration of the patient 2 weeks after the first embolization shows no filling of the treated aneurysm and a new arteriovenous shunting between the right T12 ISA and the Azygos vein (arrow). This was considered to be incidental and unrelated to the patient's clinical deterioration, but nevertheless embolized via n-butyl cyanoacrylate injection.

MRI of the spine revealed multicompartmental spinal hemorrhage, in both the subarachnoid and subdural compartments, extending from T7 down to the sacrum, and significant swelling of the lower thoracic cord and the conus medullaris (Figure 2). There was also a trace of intracranial SAH. Contrast-enhanced sequences demonstrated a small, enhanced lesion located posteriorly in the left spinal canal at the T12 level (Figure 2). This was confirmed on spinal angiography to be an ISAA of the left PSAs originating from the left T12 intersegmental artery. The patient was treated by the endovascular coil embolization of bilateral T12 intersegmental arteries to reduce direct and indirect flow to the lesion and to promote thrombosis while sparing the affected PSAs. Postprocedure angiographic acquisitions of adjacent intersegmental arteries did not demonstrate collateral supply to the aneurysm (Figure 3). A follow-up MRI performed 48 h postoperatively no longer showed the focus of enhancement at the T12 level.

Fourteen days postcoiling, after a gradual improvement in functionality, the patient developed a sudden onset of severe low back pain and acute worsening of her left lower extremity weakness. MRI of the spine was negative for new hemorrhages or new areas of contrast enhancement and demonstrated decreased cord edema. There was no evidence of acute cord infarction. A repeat spinal angiogram did not demonstrate filling of the pseudoaneurysm or new intrathecal lesions. However, there was a newly developed arteriovenous shunt between the right T12 intersegmental artery and the azygous vein. This was thought to be an incidental finding unrelated to the new symptoms and probably secondary to the prior embolization, but was nevertheless occluded by coils and glue.

The patient improved over a week and was discharged to rehab with mild residual left lower extremity paresis and paresthesias.

## Discussion

In addition to the two cases we described, we were able to find 115 cases of ruptured ISAAs in the literature, exclusively in the form of case reports and small case series (Tables 1, 2).

The mean age at the time of presentation is in the fifth decade, with female predominance (F:M = 2:1). Ruptured ISAAs are typically present with spinal and/or intracranial SAH, associated with subdural hematomas in some cases and rarely epidural hematomas. The compartment of hematomas could be related to the involved arterial segment as it traverses the various spinal compartments.

Although the precise pathophysiological mechanism of ISAAs remains unclear, case analyses with available histopathology indicate that they are the result of a dissecting process, as evidenced by the disruption of the internal elastic lamina and the absence of a three-layered architecture (1–4). This notion fits with the observed ISAAs being fusiform at a non-branching site and with the spontaneous angiographic resolution of lesions over time in some reported cases treated conservatively. The exact causes for spinal arterial dissecting aneurysms are not known and are difficult to elucidate, as histological confirmation is rarely available. Different from extracranial dissections that appear to be mainly related to trauma, connective tissue diseases, or pro-inflammatory conditions (5, 6), the etiology for intracranial or spinal arterial dissections is less well known and often remains “idiopathic”. Several suspected inciting events or predisposing factors have been proposed, such as: vascular trauma secondary to repeated vomiting episodes or sustained efforts like in the cases we presented (7), intervertebral disk herniation (8), collagenopathies (9, 10), hypertension and smoking (10–12), and inflammatory-infective causes (13, 14). While hemodynamic stresses may increase the chance for vascular remodeling and shear-stress related aneurysmal formation, their causal relationship to forming a histological dissection of the vessel wall remains elusive. It is of note, though, that spinal aneurysms have been described in patients with increased flow imposed by collateral pathways through the spinal vasculature in steno-occlusive disease (15–19) or in patients with concomitant intradural vascular malformations (20). Thus, increased hemodynamic stress may be another factor to consider when discussing the etiology of spinal arterial dissecting aneurysms.

Anatomically, lesions were evenly distributed between radiculomedullary feeders to the anterior spinal artery and radiculopial PSAs. The cervicothoracic spine was the most common site of involvement.

In all reported cases, the aneurysm was present along the ascending limb of either the radiculomedullary or a radiculopial artery and it may be hypothesized that, similar to cervical dissections being present where the mobile segment of an artery transitions into an immobile segment, in spinal aneurysms the dissection occurs at the segment where the artery is exposed to highest torsional shear stress forces.

Clinical presentations varied, depending on the extent and location of spinal hemorrhage and associated myelopathy (21). Patients with ISAAs at the upper cervical level often present with predominantly intracranial SAH and tetraparesis/plegia associated with severe headache, nausea, vomiting, and in some cases coma. Patients with ISAAs at the thoraco-lumbar levels presented with severe chest or back pain, and in some cases lower extremity paresis or plegia with paresthesia and urinary or bowel sphincter dysfunction.

ISAAs can be identified on MRI as foci of nodular enhancement along the surface of the spinal cord. Various types of spinal hemorrhages have been reported, with SAH being the most common, in some cases extending to the intracranial compartment and resulting in hydrocephalus and rarely vasospasm (22). Spinal angiography is the gold standard and necessary modality to establish a final diagnosis. ISAAs have often been described as having a “fusiform” or “tent-like” appearance on the ascending limbs of anterior or posterior radiculomedullary branches.

Of the 115 cases of ruptured ISAAs reported in the literature, 40 (35%) were managed conservatively. The majority of these (30/40, 75%) improved clinically, with spontaneous occlusion observed in those cases that underwent follow-up imaging. Seven out of 40 (18%) patients in the conservative group died; 2 (5%) from rebleeding (23, 24), 1 (3%) from severe vasospasm (24), 2 (5%) from causes not directly related to spinal hemorrhage (25, 26), and 2 (5%) from unclear causes (27, 28). Three out of 40 (7%) patients had no reported outcome.

Out of 115 patients, 65 (57%) underwent operative treatment, either by open surgery (50/65, 77%) or by endovascular intervention (15/65, 23%). Surgical resection, clipping, or wrapping of the aneurysms has been reported (1), with resection of the aneurysm being the most commonly used strategy (32/50, 64%). Complication rates for surgery were low (2/50, 4%), with 1 case of subdural hematoma (15) and another 1 case of wound infection (28). Postoperative neurological complications may be reduced by using intraoperative indocyanine green and neurophysiological monitoring (29, 30). Ischemic events are likely to be lower in patients with robust spinal arterial anastomotic networks. Embolizations of the aneurysm-bearing or -supplying artery(ies) were also performed, mostly with coiling (10, 12, 31), and in a small number of cases with particles (>150 μm) (10, 32), Onyx (7), n-butyl-2-cyanoacrylates (1), and flow-diverter (16, 17). Complication rates of endovascular treatment, in general, were higher (3/15, 20%) than those of open surgery and were mostly represented by ischemic events in the territories of the embolized vessels. All of these three cases were infarcts in the posterior inferior cerebellar artery (PICA) territory, from the treatment of ISAAs, in the presence of pre-existing vertebral artery steno-occlusion. Two from coiling (12, 15) and one from flow-diverter deployment in the vertebral artery giving rise to the aneurysm-bearing spinal artery (16). Two cases of reported deaths (2/65, 3%), one in each group, were not directly related to the spinal hemorrhage or the intervention (15, 33). The remainder of the treated patients for whom outcomes were reported (57/65, 88%) did not experience rebleeding and achieved varying degrees of neurological improvement. Management for 10 patients (10/115, 9%) was not specified in the individual case reports.

Conservative management was mostly reserved for patients with ISAAs located along the anterior radiculomedullary artery. This was due to the high risk for intervention-related ischemic cord injury and to the notion that these lesions have a higher chance of spontaneous thrombosis as compared to intracranial ones (34). Although most deaths occurred in the conservative group, rebleeding was the cause in only 5% of patients. Long-term rebleeding rates for conservatively managed ISAAs are therefore significantly lower when compared to those of their intracranial counterparts, presumably related to the smaller vessel caliber and thus diminished flow through the artery (23, 24, 34).

The choice between surgical resection and endovascular embolization depends on several factors such as the clinical status of the patient, the ISAA location, and institutional expertise. Patients were treated surgically due to neurological deterioration requiring decompression, inadequate vascular access to the aneurysm (for example, stenosis at the origin or small caliber artery), or absence of an acceptable safety margin for endovascular embolization (for example, in close proximity to the origin of the anterior spinal artery). Adequate safety margins are particularly critical when considering embolization with liquid embolic agents. Occlusion of the artery of Adamkiewicz or the entire anterior spinal artery is likely to lead to severe cord infarction, while occlusion of the PSAs is known to be less risky, given the robust collaterals. Occlusion of the intersegmental artery(ies) supplying the affected spinal arterial branch may be sufficient to decrease flow to the ISAAs and promote thrombosis in most cases. In cases of aneurysms originating from spinal radiculomedullary feeders arising directly from the vertebral arteries, flow diversion can be considered. In these cases, risks related to the procedure and the required dual antiplatelet regimen need to be leveraged against the benefits of a potential aneurysm occlusion (86).

In our experience, we believe that surgical management is the treatment of choice if additional decompression is clinically mandated, whereas an endovascular approach with parent vessel sacrifice, preferably at the origin of the radiculopial or radiculomedullary branch, is preferred in the remainder of the cases. We believe that the excellent collaterals present on the surface of the cord through the vasacorona or the rope ladder anastomoses will prevent cord ischemia, in particular in dissections of radiculopial arteries. Nevertheless, given the relatively low reported rebleeding risk, the management approach for each case needs to be tailored to the particular patient's clinical and anatomical characteristics.

## Conclusion

ISAAs are rare vascular lesions, likely dissecting in nature. Their natural history remains poorly understood; however, available data suggest that the risk of rebleeding is relatively low compared to that of their intracranial counterparts. This difference is presumably due to the lower hemodynamic stress exerted on these lesions by the reduced flow within smaller-caliber spinal vessels. Nevertheless, the potential for substantial morbidity and mortality associated with rebleeding, as highlighted in the reviewed literature, supports a proactive management approach. Whenever feasible and associated with an acceptable risk profile, surgical or endovascular parent vessel sacrifice should be considered. In cases of radiculomedullary artery dissections, the risk of treatment-related anterior spinal cord ischemia must be carefully weighed against the relatively low risk of rebleeding.
